# Recovery of Nd^3+^ and Dy^3+^ from E-Waste Using Adsorbents from Spent Tyre Rubbers: Batch and Column Dynamic Assays

**DOI:** 10.3390/molecules30010092

**Published:** 2024-12-29

**Authors:** Miguel Nogueira, Inês Matos, Maria Bernardo, Filomena Pinto, Isabel Fonseca, Nuno Lapa

**Affiliations:** 1 LAQV/REQUIMTE, Associated Laboratory for Green Chemistry, Department of Chemistry, NOVA School of Science and Technology, NOVA University Lisbon, 2829-516 Caparica, Portugal; mf.nogueira@campus.fct.unl.pt (M.N.); maria.b@fct.unl.pt (M.B.); blo@fct.unl.pt (I.F.); ncsn@fct.unl.pt (N.L.); 2 UBB-LNEG, Bioenergy and Biorrefineries Unit, Laboratório Nacional de Energia e Geologia, 1649-038 Lisboa, Portugal; filomena.pinto@lneg.pt

**Keywords:** rare earth elements, neodymium (Nd), dysprosium (Dy), spent tyre rubber, pyrolytic carbon adsorbents, adsorption, NdFeB magnets, real leachates

## Abstract

This paper investigates the use of spent tyre rubber as a precursor for synthesising adsorbents to recover rare earth elements. Through pyrolysis and CO_2_ activation, tyre rubber is converted into porous carbonaceous materials with surface properties suited for rare earth element adsorption. The study also examines the efficiency of leaching rare earth elements from NdFeB magnets using optimised acid leaching methods, providing insights into recovery processes. The adsorption capacity of the materials was assessed through batch adsorption assays targeting neodymium (Nd^3^⁺) and dysprosium (Dy^3^⁺) ions. Results highlight the superior performance of activated carbon derived from tyre rubber following CO_2_ activation, with the best-performing adsorbent achieving maximum uptake capacities of 24.7 mg·g⁻^1^ for Nd^3^⁺ and 34.4 mg·g⁻^1^ for Dy^3^⁺. Column studies revealed efficient adsorption of Nd^3^⁺ and Dy^3^⁺ from synthetic and real magnet leachates with a maximum uptake capacity of 1.36 mg·g⁻^1^ for Nd^3^⁺ in real leachates and breakthrough times of 25 min. Bi-component assays showed no adverse effects when both ions were present, supporting their potential for simultaneous recovery. Furthermore, the adsorbents effectively recovered rare earth elements from e-waste magnet leachates, demonstrating practical applicability. This research underscores the potential of tyre rubber-derived adsorbents to enhance sustainability in critical raw material supply chains. By repurposing waste tyre rubber, these materials offer a sustainable solution for rare earth recovery, addressing resource scarcity while aligning with circular economy principles by diverting waste from landfills and creating value-added products.

## 1. Introduction

Neodymium (Nd) and dysprosium (Dy) integrate the group of rare earth elements (REEs). These metals are considered critical raw materials (CRMs) by the European Union (EU) [1] and the USA Department of Energy [2] due to their economic and strategic implications, as well as the risks associated with their supply chain [3,4]. REEs play a vital role in the transition to a sustainable, low-carbon, and low-environmental-impact economy due to their unique properties [5]. REEs play a crucial role in many advanced technological applications. They are particularly vital for magnets used in electronics and communication devices. Additionally, REEs are essential in the fields of renewable energy, robotics, electric vehicles (EVs), aerospace, and defence [6]. Nd and Dy face an increasing demand due to their indispensable role in the production of high-performance permanent magnets, which further highlights the importance of sustainable strategies for their recovery [7].

E-wastes are the fastest-growing waste stream, which demands sustainable valorisation pathways for CRM recovery, reducing the EU’s dependency on external sources [8]. REE-containing permanent magnets, particularly NdFeB magnets, stand out as a promising candidate for e-waste recycling [9,10]. These magnets represent a key flow with high potential for recovering Nd and Dy [11]. Computer hard disc drives (HDDs) have been identified as one of the major candidates for REE recycling, as they represent a major resource for NdFeB magnets (up to 30 g of REEs) [11,12]. Although promising, existing recycling techniques encounter both technological and economic hurdles, necessitating the creation of sustainable and eco-friendly strategies to handle this intricate waste stream effectively [4,13].

Spent tyre rubber (STR) poses a significant global environmental threat, with its disposal escalation due to the rising production of new tyres. Approximately 17 million tons of STR are discarded annually, predominantly through landfilling and, to a lesser extent, energetic valorisation via combustion [14,15]. These disposal methods not only lead to material loss but also generate highly toxic compounds and greenhouse gases. To address this challenge, enhancing STR recycling and valorisation through innovative solutions and optimising current management policies is imperative [9,16]. Pyrolysis emerges as a promising technology for STR valorisation, converting waste into high-value products [17,18,19,20], including porous carbon materials with use in diverse applications, notably as efficient adsorbents and catalyst supports [21,22,23].

In the pursuit of sustainable and circular approaches, this research focuses on utilising porous carbons derived from STR for the recovery of REEs from e-waste. This novel method seeks to convert waste materials into valuable assets for recovering Nd and Dy from discarded NdFeB magnets. By using spent tyres as precursors for adsorbents, this approach not only tackles waste management challenges but also supports circular economy principles. As a result, STR chars and activated chars are produced and then applied as adsorbents to extract REEs from end-of-life NdFeB magnets. The study encompasses batch adsorption assays and bi-component (Dy and Nd) column adsorption assays, with a particular emphasis on employing real magnet leachates as adsorbates.

Despite the growing interest in REE recovery, studies focusing on the adsorption of REEs using pyrolytic carbon-based adsorbents remain scarce in the literature [24]. Even fewer investigations extend to multi-component systems, reflecting the inherent complexity of competitive adsorption dynamics. The transition from batch to dynamic adsorption assays is another underexplored area, leaving a significant gap in understanding scalable recovery processes. Moreover, applying these materials to real leachates, rather than simplified synthetic solutions, represents a critical innovation in this field. This study pioneers the development and application of STR-derived porous carbons in these challenging contexts, bridging these knowledge gaps and advancing sustainable strategies for REE recovery.

## 2. Results and Discussion

### 2.1. Pyrolysis Assays and Char Activation

The pyrolysis parameters used in this work were based on previous studies to maximise the char [19,20,25].

Table 1 shows that the char yield was 39.7% for A405 and 37.5% for B405. The gas yields were 9.07% and 10.5% and oil yields were 51.2% and 52.0% for char A405 and B405, respectively. It was also possible to conclude that the source of STR does not affect the pyrolysis process, as both chars (A405 and B405) presented comparable results even though they come from different sources of tyres.

These results align with expectations from previous studies and the literature reviews regarding yields at the chosen temperatures [26,27,28,29].

The activation of chars had a burn-off of 14.3% and 16.9% for A405-CO2 and B405-CO2, respectively. These results are similar to those found in the literature for activation with the same profile and activation agent [30,31,32], although they tend to be on the lower limit of the reported range results [31]. According to Hofman and Pietrzak [33], this could be attributed to the presence of ash in the chars, as it can interfere with the activation process and the reactions taking place during this process.

### 2.2. STR, Chars, and Activated Chars

**Figure 1:**
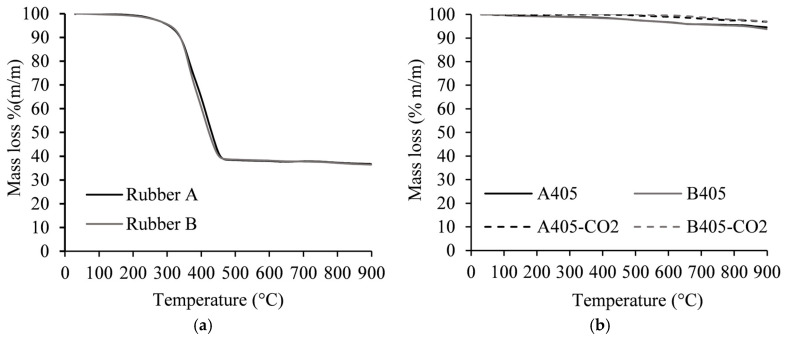
Thermogravimetric analysis. ((**a**) STR precursors; (**b**) chars and activated chars).

The thermal degradation profiles of rubbers A and B (Figure 1) are identical in both samples and show a steep mass loss of 61% wt. between 250 °C and 450 °C. Beyond this temperature range, there are no significant additional losses, resulting in a carbonaceous residue comprising 36% wt. at 900 °C. It was also observed that the source of STR does not affect the thermal decomposition process. The thermograms of the produced materials (Figure 1) revealed that both chars and activated chars have high thermal stability, showing a mass loss inferior to 10% wt. at 900 °C. Although all the materials show similar weight losses, the chars A405 and B405 show higher thermal degradations, as would be expected, since they were obtained at lower temperatures than the activated chars.

The elemental analysis (Table 2) displays an identical composition for both rubber precursors, with high carbon content, as well as a considerable amount of sulphur due to the vulcanisation procedure [34].

The produced chars retained a notably high concentration of carbon, while the levels of hydrogen and nitrogen decreased due to the thermal degradation of organic compounds during the carbonization process. This trend persists in the activated chars; however, there is a further reduction in the concentrations of hydrogen and nitrogen resulting from the activation process. On the contrary, there is an observed increase in sulphur and ash content in both chars and activated chars, attributable to the concentration effect experienced during the pyrolysis process and further accentuated during activation. This phenomenon arises from the release of volatile matter during these processes, while these compounds are retained in the solid matrix of the final products. The CAC sample exhibits a significantly lower ash content compared to the produced materials, as expected for a commercially activated carbon.

With Rubber A (9.50% *w*/*w*) containing the highest concentration of ash among both precursors, it is evident that both materials derived from said rubber (A405 and A405-CO2) have higher ash concentrations when compared to their counterparts. All the carbon samples are characterised by having an alkaline behaviour (Table 2). Nevertheless, the activated chars have higher pH_PZC_ values than their char counterparts, attributed to the increased mineral content and the modification of their surface chemistry during the activation process.

The STR samples A and B (Table 3) present a notably similar mineral composition, with both samples containing significant amounts of zinc (Zn), calcium (Ca), and iron (Fe). These elements are commonly found in various vulcanisation agents and are used as additives in the tyre manufacturing industry [9,34]. After pyrolysis and activation, these same chemical elements were detected in both chars and activated chars, albeit in higher concentrations attributed to the effect of concentration during pyrolysis. Furthermore, it is notable that Rubber B exhibits a slightly higher concentration of Zn, a characteristic also reflected in the produced materials derived from this precursor. This observation indicates that while the source of STR may not influence the pyrolysis process, its origin does indeed impact the mineral composition of the resulting materials. This variation is likely to influence the adsorption mechanism, as will be further elucidated in this work. An interesting feature is that although silicon (Si) was not detected in the rubbers and raw chars, the corresponding activated chars presented a high content of this element. A possible assumption is that Si was not solubilized in the acidic digestion, while the activation process of the chars produced a more soluble Si compound able to be quantified.

In the case of CAC, Si, Al, and Fe were its major elements, albeit in lower concentrations when compared to the produced materials, as is evident by its ash concentration [35]. The elements Cd, Se, Sn, and Ti were not detected in the samples.

**Figure 2:**
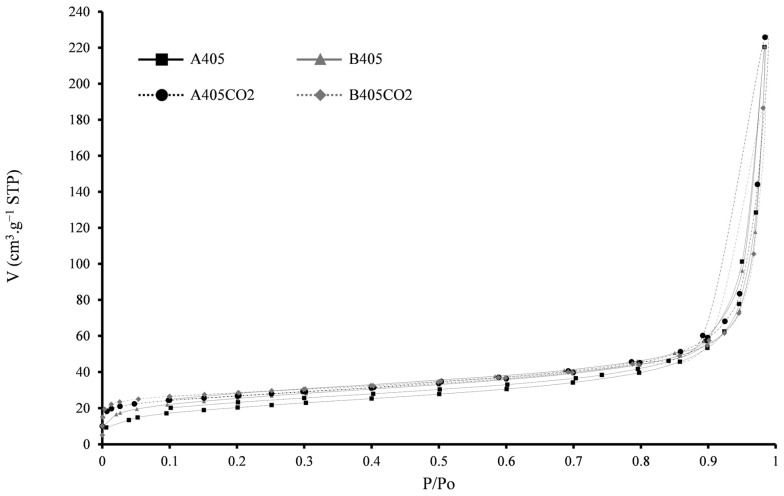
N_2_ adsorption–desorption isotherms for the produced adsorbents.

Textural characteristics of the adsorbents were acquired from N_2_ adsorption–desorption isotherms (Table 4 and Figure 2). Both chars exhibit identical surface areas (*A_BET_*), which are consistent with the values reported in the literature [21,22,32]. The activated chars A405-CO2 and B405-CO2 demonstrated a slight increase in their *A_BET_* and total pore volume in comparison with their char counterparts, attributed to the surface development from the activation with CO_2_. In the STR-derived materials, the pore volume is attributed mainly to mesopores; the contribution of microporosity increases slightly after CO_2_ physical activation. The CAC sample is characterised by a high surface area (1030 m^2^·g^−1^) with narrow mesopores and large micropores [35].

Both the produced chars and activated chars display type IV (a) isotherms [36], typically associated with mesoporous materials. Conversely, the CAC sample demonstrates a mixture of type I (b) and type IV isotherms [36], with its hysteresis linked to slit-shaped pores, a characteristic commonly observed in commercial activated carbons [35].

**Figure 3:**
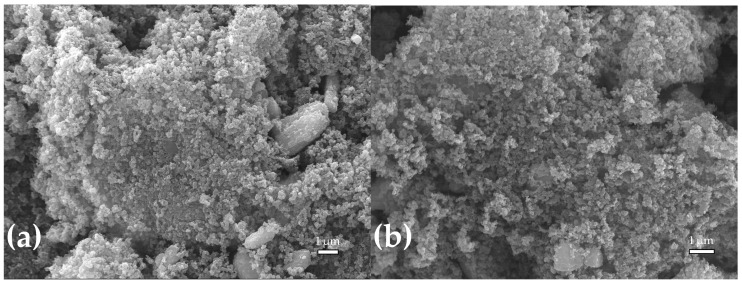

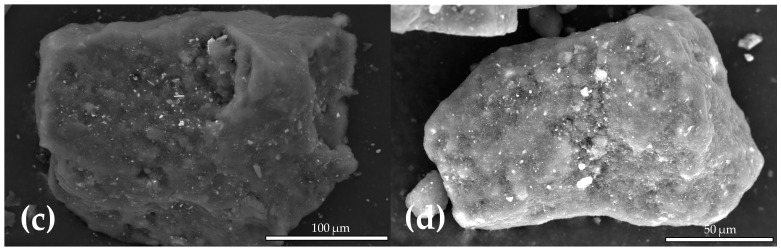
SEM images of the produced adsorbents. (**a**) A405; (**b**) B405; (**c**) A405-CO2; (**d**) B405-CO2.

The morphological structure of the produced adsorbents was analysed using SEM images (Figure 3). The surface morphology of chars A405 and B405 shows aggregates formed by disordered carbon particles, resembling structures typically observed on carbon-black surfaces and spent tyre pyrolysis chars [37]. In contrast, the ACs A405-CO2 and B405-CO2 exhibit a noticeable reduction in these aggregates, although some remain visible. This change is likely due to interactions between the adsorbent and CO_2_ at elevated temperatures during the activation process. The reduction in aggregates correlates with the development of a more defined pore structure and the degradation of the carbon matrix, resulting in smoother surfaces and increased porosity [38].

**Figure 4:**
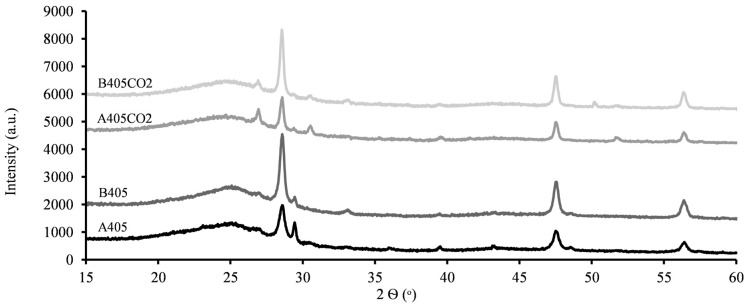
X-ray powder diffraction patterns of produced materials.

The XRPD patterns shown in Figure 4 help identify the crystalline mineral phases in the produced materials. The broad peak between 20° and 30° is indicative of the amorphous carbon characteristic of these materials. Most other peaks are attributed to zinc sulphide (ZnS) [39,40,41]. Zinc silicate (willemite—Zn_2_SiO_4_) is also detected, which results from a ZnO and SiO_2_ reaction which is used in tyre manufacturing [42,43]. Furthermore, it is possible to theorise that in the activated chars, a shift from beta-ZnS to alpha-ZnS is observable due to the elevated temperatures applied in the activation process [37,44]. Likewise, a slight increase in ZnO content can be noted, attributed to its reaction with CO_2_ [44,45], as described in Equations (1) and (2).

(1) $$Zn+{CO}_{2}↔ZnO+CO$$

(2) $$2Zns+{CO}_{2}\to 2ZnO+C+2S$$

### 2.3. Magnet Leaching

The magnet leaching processes employed in this study followed a comprehensive literature review, and optimised parameters were selected. Initially, aqua regia was selected due to its capability to fully dissolve NdFeB magnets [46,47]. Subsequentially, assays with 3M HNO_3_ were performed due to its selectivity of leaving behind iron in the precipitate [5,48]. Moreover, HNO_3_ leaching is a less demanding process in terms of energy and reagent consumption, and the literature suggests that this pathway might be a feasible approach for an industrial process [49].

According to Table 5, Fe constitutes the major element of the magnets (80.6% *w*/*w*), forming the bulk matrix. REEs were also present in the magnets, with Nd being the second most abundant element with 18.9% *w*/*w*, as it serves as the primary component for imparting magnetic properties.

Dy (1.21% *w*/*w*) and Pr (1.50% *w*/*w*) were also detected in the magnet matrix, employed as additives to enhance magnetic properties, particularly by increasing the Curie point of the magnets and enabling them to withstand higher operating temperatures without losing their capacities. Additionally, Al, B, Co, and Ni were present, albeit in trace amounts compared to REEs and Fe.

The data from the 3M HNO_3_ leaching efficiency reveals that Fe was not completely dissolved, with only 70.1% leaching. This outcome is viewed positively in the scope of this study, as its partial retention reduces competition during subsequent adsorption processes. Moreover, this partial dissolution offers the potential for recovering some Fe utilised in magnet manufacturing, thus enabling the recovery of an important by-product throughout the process. Additionally, a loss of 17.1% of Pr content from the magnets was recorded.

### 2.4. Mono-Component Batch Adsorption Assays

The four materials produced in this study were investigated in an adsorption study targeting Nd^3+^ and Dy^3+^ ions present in synthetic solutions, aiming to assess their effectiveness as REEs’ adsorbents. Mono-component adsorption tests were carried out at the original pH of the synthetic solutions of Nd^3+^ and Dy^3+^ (pH_Nd_ = 6.40, pH_Dy_ = 7.36). Speciation diagrams for both Nd and Dy indicate that the predominant species are free Nd^3+^ and free Dy^3+^ up to pH 8. Beyond this pH, these elements begin to precipitate as hydroxides. However, the final pH recorded after each assay remained below this threshold, ensuring that no precipitation phenomena interfered with the adsorption mechanisms. Furthermore, the decision to maintain the natural pH of the synthetic solution was reinforced by the literature, suggesting that reducing the solution pH does not improve the recovery efficiency of these REEs [50].

**Figure 5:**
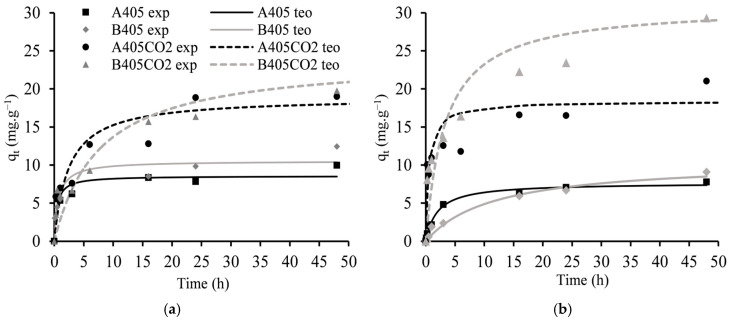
Kinetic curves for the batch adsorption assays. (**a**) Nd^3+^; (**b**) Dy^3+^.

**Figure 6:**
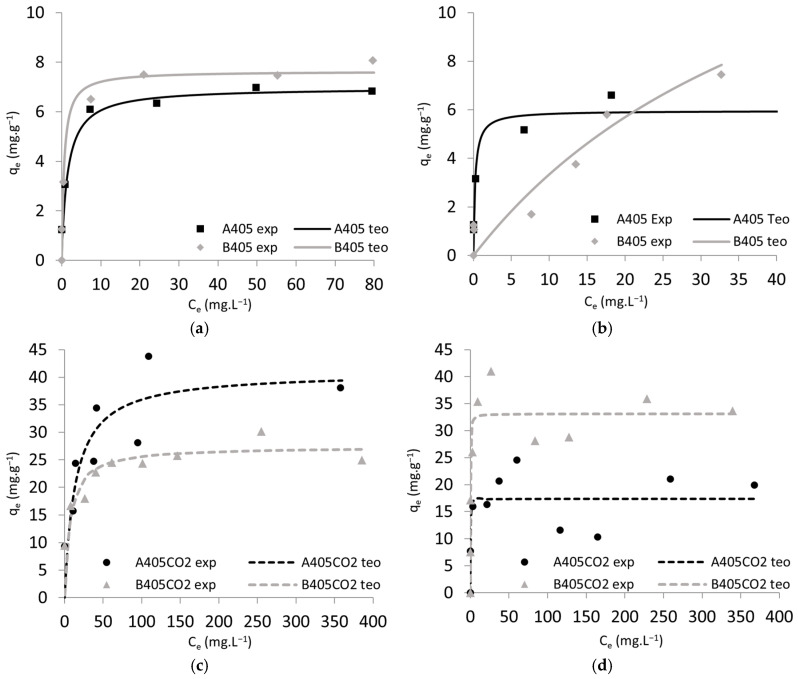
Isotherms for the batch adsorption studies. (**a**) Nd^3+^ chars; (**b**) Dy^3+^ chars; (**c**) Nd^3+^ activated chars; (**d**) Dy^3+^ activated chars. Points—experimental data; lines—Langmuir model.

Maximum uptake capacities of the produced materials, kinetic data, and isotherm data are shown in Table 6 and Figure 5 and Figure 6. Several conclusions can be drawn from these data as follows: (i) The uptake capacities of Nd^3+^ and Dy^3+^ for the same type of materials (chars or ACs) are quite similar, with no significant differences observed between both ions. However, a slightly higher affinity towards Dy^3+^ is noticeable, likely attributed to the difference in the ionic radius of both ions, with Dy^3+^ having slightly better access to the active sites on the materials [51]. (ii) Material B405 exhibits higher uptake capacities for both ions compared to char A405, a trend also observed in their activated counterparts, with B405-CO2 demonstrating superior uptake capacities over A405-CO2. (iii) The CO_2_ activation of the materials resulted in an increase in the maximum uptake capacities of both ions, with at least a two-fold improvement observed across both materials. (iv) Among all the produced materials, B405-CO2 displayed the best overall performance, with maximum uptake capacities of 24.7 mg·g^−1^ and 34.4 mg·g^−1^ for Nd^3+^ and Dy^3+^, respectively.

Understanding the sorption mechanisms involved in these processes can be very challenging in many fields of application [52]. In seeking to understand the adsorption mechanisms involved in the adsorption of Nd^3+^ and Dy^3+^, several factors must be considered [29,53].

Textural properties alone do not appear to be the determining factor, as the activated chars exhibited similar surface areas and textural properties as the chars, and the CAC sample presented a very high surface area that does not correlate with adsorption capacities [24]. This suggests that physisorption is unlikely to be the main adsorption mechanism. Furthermore, the analysis of the pH at the point of zero charge (pH_PZC_) reveals that they all exceed the pH of the REE solutions. Consequently, it is anticipated that the positively charged surface of the materials would repel Nd^3+^ and Dy^3+^, excluding the electrostatic forces as attraction mechanisms. Therefore, it is plausible that the surface chemistry of the produced materials plays a pivotal role in the adsorption mechanisms [54]. A suggested mechanism is a form of chemisorption, specifically an ion exchange mechanism [21,55,56]. A previous study conducted by the authors of this study delved deeper into these mechanisms using similar materials. Ionic exchange assays were conducted and XPS was performed on the materials before and after the adsorption of Nd^3+^ and Dy^3+^. The study found that Nd and Dy removal from the solution primarily occurred by exchanging with Zn ions present in the materials, and to a lesser extent with Ca ions. XPS analysis indicated that Nd and Dy were adsorbed in oxide form following the release of Zn and Ca [21].

The adsorption of Nd^3+^ and Dy^3+^ onto the adsorbents A405, B405, A405-CO2, and B405-CO2, was studied by using pseudo-first-order, pseudo-second-order, Langmuir, and Freundlich models (Table 6) through the minimum of the least squares method. Analysing the data from the kinetic models, it was revealed that the pseudo-second-order model offered a superior fit to the experimental data compared to the pseudo-first-order model for both ions and all materials investigated. This suggests that the adsorption process is more likely to follow chemisorption mechanisms rather than physical adsorption mechanisms, corroborating our previous findings. It is interesting to note that the kinetic constant is always higher for Nd^3+^ independent of the materials; even with slightly lower uptakes, the Nd^3+^ ion seems to present faster kinetics.

The Langmuir and Freundlich isotherm models were employed to characterise the equilibrium adsorption behaviour of Nd^3+^ and Dy^3+^ ions. The Langmuir model assumes monolayer adsorption onto a homogeneous surface, while the Freundlich model describes heterogeneous adsorption onto a surface with multiple active sites. The results indicate that the Langmuir model provides a better fit to the experimental data than the Freundlich model, implying that the adsorption of Nd^3+^ and Dy^3+^ ions onto the materials studied may occur through monolayer coverage with uniform adsorption sites. It is worth noting that the Langmuir constant KL values determined by the fitting are almost always higher for Dy^3+^, indicating a higher affinity to the material, which reflects the slight preference for this ion in the competitive sorption studies discussed next.

Beyond the detailed analysis of adsorption models and capacities, it is important to evaluate the influence of STR source on the performance of the resulting adsorbents. A comparison of the adsorption performance of materials derived from Rubber A and Rubber B revealed that despite minor differences in textural and compositional properties, their overall performance in adsorbing Nd^3^⁺ and Dy^3^⁺ was remarkably similar. For instance, B405-CO2 demonstrated slightly higher uptake capacities compared to A405-CO2, which can be attributed to minor variations in surface area and mineral composition. However, these differences were not significant enough to impact the overall feasibility or efficacy of the process. This consistency indicates that the adsorption method is robust across different STR sources, a positive indication for industrial scalability. The ability to use STR from varying origins, including blends of tyres from different suppliers, without affecting the final outcome underscores the practical applicability of this approach in real-world scenarios.

### 2.5. Column Adsorption Studies

Since the activated char B405-CO2 exhibited superior performance in the batch studies, it was decided to exclusively subject this material to dynamic adsorption studies. This focused approach allows for a more concentrated investigation into the behaviour and efficiency of this particular adsorbent under dynamic conditions.

#### 2.5.1. Mono-Component Assays

**Figure 7:**
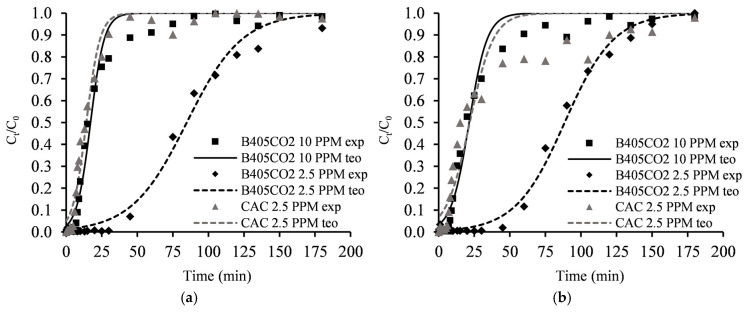
Column adsorption breakthrough curves. (**a**) Nd^3+^; (**b**) Dy^3+^ (points—experimental data; lines—theoretical data from Thomas model).

The breakthrough curves for Nd^3+^ and Dy^3+^ are depicted in Figure 7 for both B405-CO2 and CAC adsorbents.

Analysis of the data from the mono-component assays revealed that the adsorption behaviour was very similar between Nd^3+^ and Dy^3+^ for both adsorbents. Upon examining the different initial concentrations, C_0_, it became apparent that reducing C_0_ from 10 ppm to 2.5 ppm led to an increase in both the breakthrough and saturation times. For Nd^3+^, the breakthrough and saturation times improved from 7.2 min and 74.6 min to 40.2 min and 180.7 min, respectively, representing a 458% increase for breakthrough and a 142% increase for saturation. Similar behaviour was observed for Dy^3+^, where the same change in C_0_ resulted in a 511% increase in breakthrough time and a 10.9% increase in saturation time. The maximum uptake capacity for Nd^3+^ was 3.5 mg·g^−1^, while for Dy^3+^, it was 3.7 mg·g^−1^ (Table 7). These results, compared with the limited literature examples, demonstrate very promising outcomes, as the maximum uptake capacities and breakthrough times of B405-CO2 fall within the higher spectrum of reported results [57,58,59]. All experimental data fit well with the Thomas model (R^2^ values above 0.98 for all assays) (Table 8).

For benchmarking purposes, the CAC adsorbent was also evaluated, revealing that the adsorbent produced in this study exhibits much higher performance compared to the commercial alternative. The CAC adsorbent demonstrated maximum uptake capacities of 1.1 mg·g^−1^ and 1.4 mg·g^−1^ for Nd^3+^ and Dy^3+^, respectively. This corresponds to a decrease of 68.6% and 62.1% compared to B405-CO2 under the same conditions.

#### 2.5.2. Bi-Component Assays

**Figure 8:**
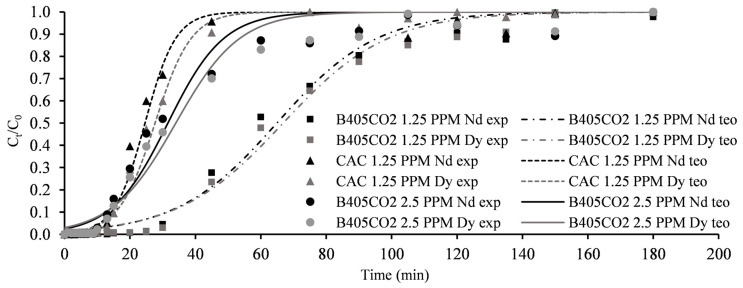
Column adsorption breakthrough curves for bi-component assays (Nd^3+^ + Dy^3+^). Points—experimental data; lines—theoretical data from Thomas model.

By analysing the data for the breakthrough curves in the bi-component assays (Figure 8), it was evident that when both Nd^3+^ and Dy^3+^ were present in the solution and competing for adsorption sites, no synergistic or antagonistic behaviours were observed.

The adsorptive behaviour of both ions was very similar, although Dy^3+^ exhibited a slight preference. This observation is consistent with the results from the mono-component assays, as well as a previous study published by the authors of this work [21], where the competitive adsorption of the same ions was investigated more extensively. Similarly to the mono-component assays, a reduction in the initial C_0_ resulted in increases in breakthrough and saturation times. Regarding maximum uptake capacities, B405-CO2 demonstrated a value of 4.3 mg·g^−1^ for both ions, divided into 2.0 for Nd^3+^ and 2.3 for Dy^3+^ (Table 7). This value is slightly higher than that recorded in the mono-component assays, possibly induced by a higher driving force due to a higher availability of adsorbate. These findings led to the conclusion that no adverse effects were observed when both REEs were present in the solution and that the adsorbents did not exhibit a higher affinity for either ion. Once again, all data fit well with the Thomas model (R^2^ values above 0.98 for all conditions studied). Similarly, the CAC adsorbent displayed lower performance compared to B405-CO2, with maximum uptake capacities of 0.5 mg·g^−1^ against 1.7 mg·g^−1^ for the material produced in this study. This represents a decrease of 70.6% in performance, mirroring the results obtained in the mono-component assays.

#### 2.5.3. Adsorption Assay with Real Leachate

The leachates used in these assays were previously treated as described in Section 3.8.3 to mimic the conditions used in the bi-component adsorption assays, with the same C_0_ for Nd^3+^, as this element presented a higher concentration in the magnets than Dy.

**Figure 9:**
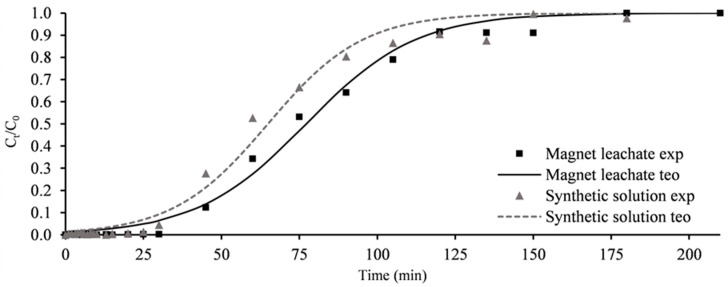
Column adsorption breakthrough curves; magnet leachates; B405-CO2 (points—experimental data; lines—theoretical data from Thomas model).

Analysing Figure 9, which illustrates the breakthrough curves for Nd^3+^ for both the magnet leachates and the synthetic solution, it became apparent that B405-CO2 exhibited a very positive performance in the magnet leachates.

Although the results were slightly inferior to those obtained with the synthetic solution, the difference was minimal. This demonstrates that in real-life scenarios, this adsorbent behaves similarly to synthetic scenarios, confirming that it is indeed feasible to use these assays as a method to further study and optimise this process in the laboratory. These results further establish that the adsorbent B405-CO2 works well when applied to real leachates.

The adsorbent B405-CO2 achieved a Nd^3+^ maximum uptake capacity of 1.36 mg·g^−1^ in the magnet leachates, a result only 20% lower than that recorded in synthetic solutions. Similarly, breakthrough and saturation times were slightly affected, with values for the magnet leachates of 25 and 129 min, respectively, representing a modest decrease of 18% and 10% compared to the synthetic solutions. The experimental data fit well to the Thomas model, with an R^2^ value of 0.99.

Overall, these results yield a positive outcome for the scope of this work, demonstrating that this waste-derived adsorbent can perform effectively in real eluates from magnets. Furthermore, the experimental data obtained in the synthetic assays align well with real-world applications, indicating the potential to promote the usage of this material and techniques beyond the laboratory. This transition to real applications could have a significant impact on promoting sustainability in the supply chain of critical raw materials and fostering a circular economy while simultaneously addressing challenges in waste management.

## 3. Materials and Methods

### 3.1. Spent Tyre Rubber—STR

STR samples were supplied by two different companies that provide recycling services for end-of-life tyres. Both samples correspond only to the rubber fraction. The fabric liner, as well as the metal reinforcement, were previously removed. Rubber A (particle size range: 0.18 to 0.60 mm) was supplied by a company that applies a cryogenic process for STR recycling mainly from small vehicles. Rubber B (particle size range: 0.60 to 0.80 mm), sourced from a company specialising in mechanical recycling, is a mixture of STR derived from both small and heavy vehicles.

### 3.2. NdFeB Magnets

The NdFeB magnets used in this research were obtained from end-of-life computer hard disc drives provided by the IT department of the NOVA School of Sciences and Technology (NOVA FCT, Lisbon, Portugal). Manual separation of the magnets from inside the hard disc drives was performed. The magnets were then demagnetized by applying temperature above their Curie point (heating rate of 10 °C·min^−1^ until 400 °C with a hold time of 1 h in a N_2_ atmosphere) [60]. Afterwards, they were manually grounded to reduce their particle size. Subsequently, they were roasted to convert the metal species into their oxides. The temperatures and roasting times were based on thermogravimetric analysis (Setaram Labsys EVO). Briefly, 25 g of magnets were heated up until 800 °C, at a heating rate of 10 °C·min^−1^, and held for 3 h. This process was performed in atmospheric air. After roasting, the magnets registered a 12.3% increase in mass due to oxide formation.

### 3.3. Pyrolysis Assays

Batch pyrolysis was performed in a 5.5 L Parr Instruments reactor with continuous stirring (Parr Instrument Company, Moline, IL, USA). After being loaded with 100 g of either Rubber A or B, the reactor was sealed, purged, and pressurised with N_2_ at 0.6 MPa. The heating rate was 5 °C·min^−1^ up to a maximum temperature of 405 °C, with a hold time of 30 min. After cooling to room temperature, the chars mixed with the pyrolysis oils were decanted. The obtained chars (A405 and B405) were extracted in a Soxhlet apparatus using hexane and acetone and then washed with deionised water. The char yield after extraction was obtained from Equation (3).

(3) $$\eta_{char}=\frac{m_{char}}{m_{tyre}}\times 100$$

where $\eta_{char}$ is the char yield (%), $m_{tyre}$ is the initial STR mass (g), and $m_{char}$ represents the obtained char mass (g).

### 3.4. Char Activation

The activation of chars was performed in a custom-made, bench-scale quartz fluidized bed reactor in vertical configuration, with PID temperature control under CO_2_. A selected amount of the char (6 g) was loaded onto the reactor, which was then purged with N_2_. A heating rate of 5 °C·min^−1^ was applied until 800 °C. At this point, the N_2_ flow was changed to CO_2_ (150 mL·min^−1^), and the sample was activated for 8 h. The resulting activated chars (ACs) were coded A405-CO2 and B405-CO2, according to the char used as the precursor.

### 3.5. Sample Characterisation

In this study, the STRs underwent several characterizations, including (i) thermogravimetric analysis (TGA), (ii) elemental analysis, (iii) mineral content determination, and (iv) ash content quantification. Chars, activated chars, and commercial activated carbon (CAC) were characterised for (i) elemental analysis, (ii) mineral content, (iii) TGA, (iv) N_2_ adsorption–desorption isotherms at 77 K, (v) pH at the point of zero charge (pH_PZC_), (vi) X-ray powder diffraction (XRPD), and (vii) scanning electron microscopy with energy-dispersive spectroscopy (SEM-EDS). The detailed characterisations are presented in the Supplementary Materials (Section S1).

### 3.6. Magnet Leaching

Magnet leaching was performed in a two-neck round-bottom flask with reflux, inside a silicon bath with temperature control and magnetic stirring. Two different leaching assays were performed as follows:

(i) Aqua regia leaching until full dissolution of the magnet was observed to acquire the full composition of the material. A total of 100 mg of powdered magnets was inserted into the flask and 60 mL of aqua regia (HNO_3_:HCl = 1:3 *m*/*m*) (HNO_3_ 65% and HCl 37%) was added. The mixture was then heated at 60 °C until complete dissolution of the magnet (40 h);
(ii) HNO_3_ 3M leaching for a set time to emulate a less demanding process in terms of energy, time, and acid. Briefly, 100 mg of powdered magnets was inserted into the flask and 60 mL of HNO_3_ 3M was added. The mixture was then heated at 60 °C for 6 h. Afterwards, the solution was filtered using ashless MCE membranes (0.22 µm). The concentrations of Al, B, Co, Dy, Fe, Nd, Ni, and Pr in both leachates were determined by ICP-AES.

### 3.7. Mono-Component Batch Adsorption Assays

Mono-component batch adsorption tests for Nd^3+^ and Dy^3+^ were performed in 20 mL sealed vials with an adsorbent/solution ratio (solid/liquid ratio) of 5 G·L^−1^. A Velp Scientifica 15-point magnetic multi-stirrer set to 300 rpm was used under a controlled temperature of 25 ± 1 °C. The solutions were filtered through 0.22 µm MCE membranes under slow vacuum conditions. Final pH values were measured with a glass pH metre (Hanna Edge) with reference solution and pH correction. The concentrations of Nd^3+^ and Dy^3+^ were analysed using ICP-AES. Nd^3+^ and Dy^3+^ recovery efficiency, η (%), and char uptake capacity $q_{exp}$ (mg·g^−1^) were determined using Equations (4) and (5).

(4) $$\eta =\frac{\left(C_{0}-C_{f}\right)}{C_{0}}\times 100$$

(5) $$q_{exp}=\frac{\left(C_{0}-C_{f}\right)}{m}\times V$$

where $C_{0}$ is the initial concentration of Nd^3+^ or Dy^3+^ (mg·L^−1^), $C_{f}$ is the final concentration (mg·L^−1^), m represents the mass of char (g), and V denotes the volume of either Nd^3+^ or Dy^3+^ solution (L). The detailed experimental procedures are presented in the Supplementary Materials (Section S2).

### 3.8. Column Adsorption Assays

The solution containing the adsorbate was introduced into the fixed-bed column (height: 10.0 cm; diameter: 0.5 cm) via an ascending flow. A peristaltic pump (Heidolph pumpdrive 5001) with multichannels was used to pump the solution into the fixed-bed column. The adsorbent was fixed by placing layers of HCl-washed silica sand and polyurethane foam at both the top and bottom. Samples were collected over time from the top end of the column. Control assays were performed to ensure that the capping materials did not adsorb any of the REEs. The performance of the adsorbents was evaluated by studying the effluent concentration over time. The resulting data were represented in a breakthrough curve, expressed as the ratio of *C_t_*/*C*_0_, representing the ratio of the adsorbate concentration in the outflow (*C_t_*) and inflow (*C*_0_) of the column, over time. The following parameters were analysed for each assay:

(i) Breakthrough time (*t_b_*)—the time taken (min) for the adsorbate concentration in the outflow to reach 5% of the inflow concentration (*C_t_∕C*_0_ = 0.05).
(ii) Saturation time (*t_s_*)—the duration (min) when the concentration of the adsorbate in the outflow equals 95% of its concentration in the inflow (*C_t_∕C*_0_ = 0.95).
(iii) Adsorbate mass retained (*m_ads_*)—the total mass (mg) of adsorbate retained up to when the saturation time is reached, which is calculated by using Equation (6).
  (6) $$m_{ads}=\frac{Q\times C_{0}}{1000}\times \int_{0}^{t_{s}}\left(1-\frac{C_{t}}{C_{0}}\right)dt$$
  where *Q* signifies the flow rate of adsorbate’s solution (mL·min^−1^), *C*_0_ represents the initial concentration of the adsorbate in the inflowing solution (mg·L^−1^), and *C_t_* stands for the concentration of the adsorbate in the outflow at time *t* (mg·L^−1^).
(iv) Removal efficiency (*η*)—the ratio between the total mass of adsorbate retained by the adsorbent and the total mass of adsorbate inserted into the column, expressed as a percentage (Equation (7)).
  (7) $$\eta =\frac{m_{ads}}{m_{total}}\times 100$$
  where *m_total_* is the total mass (mg) of adsorbate inserted into the column (Equation (8)) and *m_ads_* has the same meaning and unit as for the previous equation (Equation (6)).
  (8) $$m_{total}=\frac{Q\times C_{0}\times t_{s}}{1000}$$
  where the variables have the same meaning and units as for the previous equations.
(v) Total uptake capacity (*q_total_*)—the maximum amount of adsorbate removed by the adsorbent inside the column within the saturation time (*t_s_*) (Equation (9)).
  (9) $$q_{total}=\frac{m_{ads}}{m_{AC}}$$
  where *m_AC_* is the total mass of adsorbent (mg) inside the column.

#### 3.8.1. Mono-Component Dynamic Adsorption Assays

Mono-component dynamic adsorption assays were performed with either Nd^3+^ or Dy^3+^ synthetic solutions, using B405-CO2 or CAC as adsorbents. The experimental conditions were as follows: (i) 100 mg of adsorbent (*m_AC_*); (ii) flow rate (*Q*) of 1.5 mL·min^−1^; (iii) running time (*t*) of 180 min; (iv) temperature of 23 °C; and (v) initial concentrations of Nd^3+^ or Dy^3+^ (*C*_0_) of 10 and 2.5 mg·L^−1^ for B405-CO2 and 2.5 mg·L^−1^ for CAC.

#### 3.8.2. Bi-Component Dynamic Adsorption Assays

Bi-component dynamic adsorption assays were performed using a mixture of both Nd^3+^ and Dy^3+^ synthetic solutions, using B405-CO2 or CAC as adsorbents. The experimental conditions were as follows: (i) mass of adsorbent (*m_AC_*): 100 mg; (ii) flow rate (*Q*): 1.5 mL·min^−1^; (iii) running time (*t*): 180 min; (iv) temperature: 23 °C; and (v) initial concentrations (*C*_0_) of both Nd^3+^ and Dy^3+^ (*C_O_Nd_* + *C*_0*_Dy*_): 2.5 + 2.5 and 1.25 + 1.25 mg·L^−1^.

#### 3.8.3. Real Leachate Assays

To evaluate the adsorbents’ performance on real leachate, the HNO_3_ 3M magnet leachate (Section 3.6) was used. Before the dynamic adsorption assays, the leachate was treated as follows: (i) pH adjustment to 5.1 with NaOH 6M to precipitate iron; (ii) filtration using ashless MCE membranes (0.22 µm); and (iii) dilution with Milli-Q water until the desired concentration of 1.25 mg·L^−1^ Nd^3+^. The experimental conditions used during this assay were the following: (i) *m_AC_*: 100 mg of adsorbent; (ii) *Q*: 1.5 mL·min^−1^; (iii) *t*: 210 min; (iv) temperature: 23 °C; and (v) initial concentrations of Nd (*C*_0*_Nd*_) 1.25, Al (*C*_0*_Al*_) 0.02, B (*C*_0*_B*_) 0.13, Co (*C*_0*_Co*_) 0.08, Dy (*C*_0*_Dy*_) 0.10, Fe (*C*_0*_Fe*_) < 0.001, Ni (*C*_0*_Ni*_) < 0.007, and Pr (*C*_0*_Pr*_) < 0.01 mg·L^−1^.

#### 3.8.4. Breakthrough Modelling

The breakthrough data were fitted to Thomas’ nonlinear model [61] (Equation (10)).

(10) $$\frac{C_{t}}{C_{0}}=\frac{1}{1+e^{\left(k_{th}\cdot q_{0}\cdot \frac{m_{AC}}{Q}\right)-\left(k_{th}\cdot C_{0}\cdot t\right)}}$$

where $C_{t}$ (mg·L^−1^) represents the concentration of the adsorbate in the column outflow at time t (min), while $C_{0}$ (mg·L^−1^) is the concentration of the adsorbate in the column inflow. $k_{th}$ (mL·min^−1^·mg^−1^) is the Thomas rate constant, $q_{0}$ (mg·g^−1^) is the maximum uptake capacity of the adsorbent, $m_{AC}$ (g) denotes the mass of the adsorbent in the column, and Q (mL·min^−1^) is the flow rate of the adsorbate’s solution.

The model fitting was performed using the SOLVER plugin for MS Excel using the minimum sum of the least squares method (Equation (11)).

(11) $$Min\left[\sum Leastsquares\right]=Min\left[\sum {\left(q_{exp}-q_{teo}\right)}^{2}\right]$$

where *q_exp_* is the experimental uptake capacity (mg·g^−1^) and *q_teo_* is the theoretical uptake capacity (mg·g^−1^) calculated from Thomas’ model.

## 4. Conclusions

This study demonstrates the successful transformation of spent tyre rubbers into valuable waste-derived adsorbents through pyrolysis and activation processes. The resulting materials exhibited remarkable efficacy in adsorbing Nd^3+^ and Dy^3+^ from both synthetic solutions and real magnet leachates.

The synthesised waste-derived adsorbents, particularly the activated char B405-CO2, displayed superior performance compared to commercially available activated carbon (CAC), underscoring the potential of STR-derived materials in environmental remediation and resource recovery efforts.

Moreover, dynamic column adsorption studies revealed consistent adsorption behaviour across varying solution compositions and conditions, reaffirming the versatility and robustness of these waste-derived adsorbents. The close correlation between laboratory findings and real-world eluate suggests the scalability and practical applicability of the developed adsorption process.

This study primarily focuses on Nd^3^⁺ and Dy^3^⁺; however, the adsorbents’ textural properties, such as their pore structure, surface area, and surface chemistry, suggest a potential for broader applicability. Given the similar ionic radii and chemical behaviour of other REEs and certain heavy metals, these materials may be effective for a wider range of adsorption targets.

While this study demonstrates the technical feasibility of producing STR-derived adsorbents and their efficacy in rare earth recovery, further investigation is required to evaluate their economic viability and environmental impact. Factors such as energy consumption, emissions from pyrolysis and activation, and cost–benefit analyses need to be assessed to fully understand and optimise the sustainability of the process. Addressing these aspects in future research will be essential for facilitating large-scale implementation and strengthening the argument for the eco-friendliness of STR-derived adsorbents. Such investigations, while beyond the scope of this work, will provide valuable insights for maximising the practical and environmental benefits of this approach.

In summary, this study highlights the transformative potential of repurposing spent tyre rubber into valuable waste-derived materials for addressing secondary raw material valorisation and driving innovation across the value chains of tyres and electronic equipment.

## Figures and Tables

**Table 1:** Pyrolysis assay yields and activation burn-off rates.

		η (% *w*/*w*)		
		Char	Oil	Gas
Pyrolysis	A405	39.8	51.2	9.07
	B405	37.5	52.0	10.5
		Burn-off (% *w*/*w*)		
Activation	A405-CO2		14.3	
	B405-CO2		16.9	

**Table 2:** Elemental analysis, ash content, and pH_PZC_ of samples (as-received basis).

Sample	C	H	N	S	Ash	pH_PZC_
	(% *w*/*w*)					
Rubber A	79.2	7.07	0.40	1.64	9.50	n.d.
Rubber B	83.4	7.60	0.40	2.04	8.70	n.d.
A405	71.3	0.71	0.28	2.51	36.2	7.4
B405	79.1	0.86	0.33	3.94	31.4	6.7
A405-CO2	70.3	0.16	0.23	2.90	38.8	8.5
B405-CO2	76.3	0.14	0.29	3.70	31.7	7.8
CAC	86.3	0.47	<0.20	0.57	5.70	9.1

n.d.: not determined.

**Table 3:** Mineral composition of precursors, chars, and activated carbons (mg·g^−1^; x ± o).

Element	Rubber A	Rubber B	A405	B405	A405-CO2	B405-CO2
Al	<0.002	<0.001	<0.002	<0.002	3.92 ± 0.05	2.93 ± 0.08
Ba	0.202 ± 0.022	<4 × 10^−5^	<4 × 10^−5^	<4 × 10^−5^	0.029 ± 0.001	0.022 ± 0.001
Ca	13.0 ± 3.9	6.38 ± 0.18	21.9 ± 0.5	11.9 ± 0.8	16.56 ± 0.56	5.21 ± 0.19
Cr	0.002 ± 0.001	0.006 ±0.003	0.008 ± 0.001	0.010 ± 0.003	0.013 ± 0.001	0.011 ± 0.001
Cu	0.473 ± 0.049	1.02 ±0.17	0.318 ± 0.014	1.63 ± 0.03	0.23 ± 0.05	0.67 ± 0.03
Fe	2.18 ± 0.55	4.25 ± 0.27	4.96 ± 0.09	8.75 ± 0.15	4.59 ± 0.07	5.87 ± 0.21
K	<0.077	<0.078	<0.08	<0.08	3.15 ± 0.02	0.54 ± 0.01
Mg	0.815 ± 0.239	0.870 ± 0.105	1.79 ± 0.09	1.74 ± 0.01	1.66 ± 0.03	1.15 ± 0.05
Mn	0.029 ± 0.008	0.025 ± 0.002	0.052 ± 0.006	0.059 ± 0.002	0.054 ± 0.002	0.029 ± 0.001
Mo	<4 × 10^−4^	<4 × 10^−4^	0.036 ± 0.001	0.120 ± 0.006	0.021 ± 0.003	0.043 ± 0.003
Na	<1 × 10^−4^	<1 × 10^−4^	<1 × 10^−4^	<1 × 10^−4^	0.010 ± 0.001	0.001 ± 2 × 10^−5^
Ni	0.004 ± 0.001	0.004 ± 0.001	0.012 ± 0.001	0.016 ± 0.001	0.012 ± 0.001	0.014 ± 0.002
Pb	0.081 ± 0.027	0.043 ± 0.001	0.118 ± 0.003	0.112 ± 0.003	0.041 ± 0.002	0.036 ± 0.001
Si	<0.002	<0.002	<0.002	<0.002	105.0 ± 13.2	39.16 ± 7.18
Zn	29.2 ± 0.2	38.6 ± 5.4	69.6 ± 1.8	93.5 ± 0.8	49.55 ± 0.10	67.54 ± 3.97

**Table 4:** Textural characteristics of the produced carbons.

Sample	A_BET_	V_total_	V_micro_	V_meso_
	(m^2^·g^−1^)	(cm^3^·g^−1^)		
A405	73	0.133	0.008	0.125
B405	90	0.125	0.016	0.109
A405-CO2	95	0.140	0.020	0.120
B405-CO2	104	0.123	0.026	0.097
CAC	1030	0.56	0.30	0.26

**Table 5:** Magnet composition and 3M HNO_3_ leaching efficiency.

	Al	B	Co	Dy	Fe	Nd	Ni	Pr
Magnet composition (*w*/*w* %)	0.41	0.69	0.59	1.21	80.6	18.9	0.03	1.50
3M HNO_3_ leaching efficiency (%)	100	100	100	100	70.1	100	100	82.9

**Table 6:** Kinetics and isotherm data for the batch adsorption studies. Pseudo-first-order model, pseudo-second-order model, Langmuir model, and Freundlich model.

		A405	B405	A405-CO2	B405-CO2
		Pseudo-First-Order			
Nd^3+^	q_e,ajust_ (mg·g^−1^)	7.04	8.89	17.62	21.26
	k_ads ajust_ (h^−1^)	17.79	2.00	0.26	0.10
	R^2^	0.71	0.70	0.86	0.92
Dy^3+^	q_e,ajust_ (mg·g^−1^)	7.04	8.94	18.13	27.29
	k_ads ajust_ (h^−1^)	0.42	0.07	1.30	0.24
	R^2^	0.98	0.97	0.67	0.85
		Pseudo-Second-Order			
Nd^3+^	q_e,ajust_ (mg·g^−1^)	8.56	10.53	18.89	24.07
	k_ads ajust_ (h^−1^)	0.26	0.14	0.22	0.01
	R^2^	0.91	0.91	0.89	0.94
Dy^3+^	q_e,ajust_ (mg·g^−1^)	7.67	10.54	18.41	30.97
	k_ads ajust_ (h^−1^)	0.07	0.01	0.10	0.01
	R^2^	0.99	0.98	0.76	0.92
		Langmuir			
Nd^3+^	q_max_ (mg·g^−1^)	6.97	7.63	40.91	27.36
	K_L_ (L·mg^−1^)	0.66	1.68	0.07	0.14
	R^2^	0.99	0.98	0.70	0.86
Dy^3+^	q_max_ (mg·g^−1^)	5.96	19.29	17.33	33.13
	K_L_ (L·mg^−1^)	4.38	0.02	8.79	7.32
	R^2^	0.96	0.93	0.66	0.90
		Freundlich			
Nd^3+^	K_F_ (mg^1−1/n^·L^1/n^·g^−1^)	5.18	3.97	12.47	13.11
	1·n^−1^	0.06	0.17	0.23	0.13
	R^2^	0.95	0.97	0.69	0.95
Dy^3+^	K_F_ (mg^1−1/n^·L^1/n^·g^−1^)	3.50	0.53	13.02	22.13
	1·n^−1^	0.18	0.77	0.07	0.09
	R^2^	0.95	0.93	0.61	0.78

**Table 7:** Column adsorption assay parameters.

	C_0_ (mg·L^−1^)		B405-CO2	CAC
		Single component		
Nd^3+^	10	t_r_ (min)	7.20	-
		t_s_ (min)	74.6	-
		q_max_ (mg·g^−1^)	2.93	-
	2.5	t_r_ (min)	40.20	5.4
		t_s_ (min)	180.7	125.7
		q_max_ (mg·g^−1^)	3.48	1.14
Dy^3+^	10	t_r_ (min)	7.90	-
		t_s_ (min)	102.4	-
		q_max_ (mg·g^−1^)	3.69	-
	2.5	t_r_ (min)	48.30	23.8
		t_s_ (min)	113.5	166.3
		q_max_ (mg·g^−1^)	1.87	1.40
		Bi-component		
Nd^3+^ + Dy^3+^				
Nd^3+^	2.5	t_r_ (min)	11.90	-
		ts (min)	180.5	-
		q_max_ (mg·g^−1^)	1.98	-
Dy^3+^	2.5	t_r_ (min)	12.90	-
		t_s_ (min)	181.7	-
		q_max_ (mg·g^−1^)	2.30	-
Nd^3+^	1.25	t_r_ (min)	30.40	11.9
		t_s_ (min)	144.2	180.5
		q_max_ (mg·g^−1^)	0.80	0.20
Dy^3+^	1.25	t_r_ (min)	31.30	12.8
		t_s_ (min)	120.5	30.5
		q_max_ (mg·g^−1^)	0.90	0.30
		Real Leachate		
Nd^3+^	1.25	t_r_ (min)	35.90	-
		t_s_ (min)	161.8	-
		q_max_ (mg·g^−1^)	1.36	-

**Table 8:** Column adsorption assays data and Thomas model.

	C_0_ (mg·L^−1^)		B405-CO2	CAC
		Single component		
Nd^3+^	10	q_0_ (mg·g^−1^)	2.24	-
		kth (mL·min^−1^·mg^−1^)	23.4	-
		R^2^	0.98	-
	2.5	q_0_ (mg·g^−1^)	2.65	0.43
		kth (ml·min^−1^·mg^−1^)	24.5	102.9
		R^2^	0.99	0.98
Dy^3+^	10	q_0_ (mg·g^−1^)	2.74	-
		kth (mL·min^−1^·mg^−1^)	18.2	-
		R^2^	0.98	-
	2.5	q_0_ (mg·g^−1^)	2.54	0.72
		kth (ml·min^−1^·mg^−1^)	29.7	51.4
		R^2^	0.99	0.94
		Bi-component		
Nd^3+^ + Dy^3+^				
Nd^3+^	2.5	q_0_ (mg·g^−1^)	0.67	-
		kth (mL·min^−1^·mg^−1^)	79.0	-
		R^2^	0.98	-
Dy^3+^	2.5	q_0_ (mg·g^−1^)	0.74	-
		kth (mL·min^−1^·mg^−1^)	69.7	-
		R^2^	0.98	-
Nd^3+^	1.25	q_0_ (mg·g^−1^)	0.71	0.39
		kth (mL·min^−1^·mg^−1^)	86.5	189.5
		R^2^	0.99	0.99
Dy^3+^	1.25	q_0_ (mg·g^−1^)	0.80	0.48
		kth (mL·min^−1^·mg^−1^)	77.2	148.5
		R^2^	0.99	0.99
		Real Leachate		
Nd^3+^	1.25	q_0_ (mg·g^−1^)	1.28	-
		kth (mL·min^−1^·mg^−1^)	51.1	-
		R^2^	0.99	-

## Data Availability

All necessary data are made available in the paper and Supplementary Materials. Additional data cannot be made available for confidential reasons, as this work was supported by three companies.

## Supplementary Materials

The following supporting information can be downloaded at: https://www.mdpi.com/article/10.3390/molecules30010092/s1, Section S1—Sample characterisation; Section S2—Mono-component batch adsorption assays.
