# Supplementary material for: Recovery of Nd3+ and Dy3+ from E-Waste Using Adsorbents from Spent Tyre Rubbers: Batch and Column Dynamic Assays
Source: Molecules. 2024 Dec 29;30(1):92. doi: 10.3390/molecules30010092 (PMC11722004; doi:10.3390/molecules30010092)
Supplement: Supplementary file 1 [file molecules-30-00092-s001.zip › molecules-3368255-supplementary.pdf]

molecules

MDPI

*Article*

# Recovery of $Nd^{3+}$ and $Dy^{3+}$ from E-Waste Using Adsorbents from Spent Tyre Rubbers: Batch and Column Dynamic Assays

**Miguel Nogueira [1], Inês Matos [1,*], Maria Bernardo [1], Filomena Pinto [2], Isabel Fonseca [1] and Nuno Lapa [1]**

1 LAQV/REQUIMTE, Associated Laboratory for Green Chemistry, Department of Chemistry, NOVA School of Science and Technology, NOVA University Lisbon, 2829-516 Caparica, Portugal; mf.nogueira@campus.fct.unl.pt (M.N.); maria.b@fct.unl.pt (M.B.); blo@fct.unl.pt (I.F.); ncsn@fct.unl.pt (N.L.)
2 UBB-LNEG, Bioenergy and Biorrefineries Unit, Laboratório Nacional de Energia e Geologia, 1649-038 Lisboa, Portugal; filomena.pinto@lneg.pt
* Correspondence: ines.matos@fct.unl.pt

# Supplementary Materials

## S1. Sample Characterisation

### 1.1. *Elemental analysis and ash content*

Elemental analysis (CNHS) was quantified in a Thermo Finnigan-CE Instruments Flash EA 1112 CHNS Analyzer following the standards ASTM D5373 (C, H, N) and ASTM D4239 (S).

Ash content was determined by gravimetry. Samples were heated at 5 °C.min$^{-1}$ in atmospheric air and held for 3 h at 750 °C. The residue left was counted as ashes.

### 1.2. *Mineral content*

Mineral content was determined in two steps following standard EN-15290 (European Committee for Standardization, 2011) as follows: (i) microwave-assisted acid digestion (Milestone Ethos 1600) with 3 mL $H_2O_2$ (30 % v/v), 8 mL $HNO_3$ (65% v/v), and 2 mL HF (40% v/v), followed by a neutralisation step with 20 mL of $H_3BO_3$ (4% w/v) and (ii) the quantification of 17 metals and metalloids by inductively coupled plasma atomic emission spectroscopy (ICP-AES) (Horiba Jobin-Yvon Ultima).

### 1.3. *$pH_{PZC}$*

The pH at the point of zero charge was calculated by the point drift method (Dias et al., 2018): (i) 0.1 M NaCl solutions with pH values between 2 and 12 were prepared (NaOH and HCl were used for pH adjustments); (ii) 0.1 g of either char or AC was added to the previous solutions; (iii) the solutions were then stirred at 300 rpm for 24 h; and (iv) the solutions were filtered and the final pH of each was measured. The $pH_{PZC}$ value of each material corresponds to the plateau of the curve $pH_{final}$ *vs* $pH_{initial}$.

### 1.4. *Textural analysis*

To evaluate the textural properties of the carbon samples, $N_2$ adsorption–desorption isotherms at 77 K were acquired and the following parameters were quantified: (i) apparent surface area ($S_{BET}$) by using the Brunauer–Emmett–Teller (BET) equation; (ii) total pore volume ($V_{total}$) determined by the amount of nitrogen adsorbed at the relative pressure $P/P_o$ = 0.99; (iii) micropore volume ($V_{micro}$) using the t-plot method; and (iv) mesopore volume ($V_{meso}$) by the difference between $V_{total}$ and $V_{micro}$.

### 1.5. *SEM-EDS*

 https://doi.org/10.3390/molecules30010092

Surface structure and morphology were analysed by SEM-EDX using JEOL 7001F with an Oxford light elements EDX detector and EBSD detector equipped with a field emission gun with Schottky emission equipped with secondary and backscattered electron detectors.

*1.6. Thermogravimetric analysis*

For the rubber samples, chars, and activated chars, TGA was performed in a Setaram Labsys instrument, EVO model, with a 20 $cm^3.min^{-1}$ argon flow and a heating rate of 5 ºC.$min^{-1}$ up to 900 °C. For the magnet samples, the same apparatus was used, but the maximum temperature was 1000 °C and atmospheric air was used.

*1.7. XRPD*

The XRPD diffractograms were acquired using a benchtop X-ray diffractometer (Rigaku MiniFlex II) with a Cu X-ray tube (30 kV/15 mA) by continuous scanning from 15° to 80° (2Θ) with a step size of 0.01° and a scan speed of 2°.$min^{-1}$.

## S2. Mono-component batch adsorption assays

*2.1. $Nd^{3+}$ and $Dy^{3+}$ synthetic solutions*

$Nd^{3+}$ stock solution (1000 mg.$L^{-1}$) was prepared by dissolution of neodymium(III) nitrate hexahydrate salts ($Nd(NO_3)_3{\cdot}6H_2O$ of 99.9% purity, Sigma-Aldrich) in ultrapure water (Millipore, Milli Q Academic). $Dy^{3+}$ stock solution (1000 mg.$L^{-1}$) was prepared using dysprosium(III) nitrate hydrate salts ($Dy(NO_3)_3{\cdot}xH_2O$ of 99.9% purity, Sigma-Aldrich) in ultrapure water. Lower concentrations were obtained by diluting the stock solutions as needed with ultrapure water.

*2.2. Kinetic Study*

The experimental conditions for the kinetic study were the same for both $Nd^{3+}$ and $Dy^{3+}$ and were as follows: (i) initial $Nd^{3+}$ or $Dy^{3+}$ concentration: 100 mg.$L^{-1}$; (ii) adsorbent/solution ratio (solid/liquid ratio): 5 g.$L^{-1}$; (iii) contact time: between 0.08 (5 minutes) and 72 hours; and (iv) agitation: 300 rpm in a magnetic stirrer.

The experimental results were modelled through pseudo-first-order (Ho, 2006) (Eq. S1) and pseudo-second-order kinetic models (Ho, 2006) (Eq. S2).

$$q_t = q_e \times [1 - e^{-k_f \times t}] \quad \text{(Eq. S1)}$$

$$q_t = \frac{k_s \times q_e^2 * t}{1 + q_e \times k_s \times t} \quad \text{(Eq. S2)}$$

where $q_t$ is the uptake capacity of $Nd^{3+}$ or $Dy^{3+}$ in time $t$ (mg.$g^{-1}$), $q_e$ is the uptake capacity in equilibrium (mg.$g^{-1}$), $k_f$ is the pseudo-first-order constant ($h^{-1}$), $k_s$ is the pseudo-second-order constant (g.$mg^{-1}$.$h^{-1}$), and $t$ is the contact time (h).

*2.3. Equilibrium studies—Adsorption isotherms*

To acquire the adsorption isotherms, the following experimental conditions were used: (i) initial concentrations: 10, 20, 40, 50, 60, 80, 100, 150, 200 mg.$L^{-1}$; (ii) S/L ratio: 5 g.$L^{-1}$; (iii) contact time: 72 h; and (iv) agitation: 300 rpm in a magnetic stirrer.

The acquired data were fitted to Langmuir's model (Al-Ghouti & Da'ana, 2020; Foo & Hameed, 2010) (Eq. S3) and Freundlich's model (Al-Ghouti & Da'ana, 2020; Foo & Hameed, 2010) (Eq. S4).

$$q_e = \frac{Q^0 \times b \times C_e}{1 + b \times C_e} \quad \text{(Eq. S3)}$$

$$q_e = K_F \times C_e^{1/n} \quad \text{(Eq. S4)}$$

where $q_e$ is the $Nd^{3+}$ or $Dy^{3+}$ uptake capacity in equilibrium ($mg.g^{-1}$), $Q^0$ is the monolayer uptake capacity ($mg.g^{-1}$), $b$ is the Langmuir's constant ($L.mg^{-1}$), $C_e$ is the $Nd^{3+}$ or $Dy^{3+}$ concentration in equilibrium ($mg.L^{-1}$), $K_F$ is the Freundlich's constant, which represents the relative uptake capacity (**$mg^{1-1/n}$ $L^{1/n}$ $g^{-1}$**), and $1/n$ is the adsorption intensity (dimensionless).

## 2.4. *Modelling*

The kinetic and adsorption isotherm data were fitted to the chosen models by using the SOLVER function of MS EXCEL. The adjustments were performed through the minimization of the sum of least squares (Eq. S5).

$$\sum Least\ squares = \sum (q_{exp} - q_{calc})^2 \quad \text{(Eq. S5)}$$

where $q_{exp}$ is the experimental $Nd^{3+}$ or $Dy^{3+}$ uptake capacity ($mg.g^{-1}$) and $q_{calc}$ is the modelled $Nd^{3+}$ or $Dy^{3+}$ uptake capacity ($mg.g^{-1}$).

**Disclaimer/Publisher's Note:** The statements, opinions and data contained in all publications are solely those of the individual author(s) and contributor(s) and not of MDPI and/or the editor(s). MDPI and/or the editor(s) disclaim responsibility for any injury to people or property resulting from any ideas, methods, instructions or products referred to in the content.
